# Pharmacogenomics for neurodegenerative disorders - a focused review

**DOI:** 10.3389/fphar.2024.1478964

**Published:** 2024-12-20

**Authors:** S. Rehan Ahmad, Md. Zeyaullah, Mohammad Suhail Khan, Abdullah M. AlShahrani, Abdelrhman A. Galaleldin Altijani, Haroon Ali, Adam Dawria, Ali Mohieldin, Mohammad Shane Alam, Awad Osman Abdalla Mohamed

**Affiliations:** ^1^ Hiralal Mazumdar Memorial College for Women, West Bengal State University, Kolkata, India; ^2^ Department of Basic Medical Science, College of Applied Medical Sciences, Khamis Mushait Campus, King Khalid University (KKU), Abha, Saudi Arabia; ^3^ Department of Public Health, College of Applied Medical Sciences, Khamis Mushait Campus, King Khalid University (KKU), Abha, Saudi Arabia; ^4^ Department of Medical Laboratory Technology, College of Applied Medical Sciences, Jazan University, Jazan, Saudi Arabia; ^5^ Department of Anaesthesia Technology, College of Applied Medical Sciences, Khamis Mushait Campus, King Khalid University (KKU), Abha, Saudi Arabia

**Keywords:** pharmacogenomics, neurodegenerative disorders, Alzheimer’s disease, Parkinson’s disease, Huntington’s disease, amyotrophic lateral sclerosis, genome-wide association studies, next-generation sequencing

## Abstract

Neurodegenerative disorders such as Alzheimer’s disease (AD), Parkinson’s disease (PD), Huntington’s disease (HD), and amyotrophic lateral sclerosis (ALS) are characterized by the progressive degeneration of neuronal structure and function, leading to severe cognitive and motor impairments. These conditions present significant challenges to healthcare systems, and traditional treatments often fail to account for genetic variability among patients, resulting in inconsistent therapeutic outcomes. Pharmacogenomics aims to tailor medical treatments based on an individual’s genetic profile, thereby improving therapeutic efficacy and reducing adverse effects. This focused review explores the genetic factors influencing drug responses in neurodegenerative diseases and the potential of pharmacogenomics to revolutionize their treatment. Key genetic markers, such as the APOE ε4 allele in AD and the CYP2D6 polymorphisms in PD, are highlighted for their roles in modulating drug efficacy. Additionally, advancements in pharmacogenomic tools, including genome-wide association studies (GWAS), next-generation sequencing (NGS), and CRISPR-Cas9, are discussed for their contributions to personalized medicine. The application of pharmacogenomics in clinical practice and its prospects, including ethical and data integration challenges, are also examined.

## Introduction

Neurodegenerative illnesses such as Alzheimer’s disease (AD), Parkinson’s disease (PD), Huntington’s disease (HD), and amyotrophic lateral sclerosis (ALS) are distinguished by the gradual deterioration of neurones’ structure and function (Ballard et al., 2011; Guerini et al., 2015). These incapacitating ailments result in profound cognitive and motor impairment, significantly affecting the overall quality of life and presenting enormous obstacles to healthcare systems (Faraco et al., 2018; Scarmeas and Stern, 2004). Conventional treatment methods frequently fail to consider the genetic diversity among individuals, resulting in inconsistent reactions to treatments. Pharmacogenomics seeks to customize medication treatments according to an individual’s genetic composition, potentially improving effectiveness and reducing adverse side effects (Collins and Varmus, 2015).

Neurodegenerative illnesses are intrinsically intricate, with several causes that include genetic, environmental, and behavioural variable (Saido and Iwata, 2001; Zhang and Tropea, 2014). The progressive nature of many diseases complicates the search for viable treatments. Present therapeutic approaches prioritize alleviating symptoms rather than modifying or curing the condition, frequently resulting in less-than-optimal results. Moreover, the variation in genetic variety across patients leads to considerable differences in how they respond to drugs, highlighting the need for a more individualized approach to therapy (Zlokovic, 2011). Pharmacogenomics investigates the impact of an individual’s genetic composition on their reaction to medications, providing a route to more tailored and efficient therapies (Thies and Bleiler, 2013). Pharmacogenomics seeks to enhance the treatment of neurodegenerative disorders by customizing drugs according to genetic profiles. This approach intends to maximize the effectiveness of therapy while minimizing adverse side effects, ultimately enhancing the overall management of these conditions (Sperling et al., 2011a; Sperling et al., 2011b; Vassar and Citron, 2000).

## Genetic factors affecting drug response

### Alzheimer’s disease

AD is an intricate neurological ailment that is affected by numerous hereditary variables (Zs-Nagy, 2010; Montine et al., 2012). The APOE gene is a highly influential genetic factor in determining the risk and course of AD (Shanker et al., 2010; Lovestone and Harrington, 2006; Scheltens and Van der Flier, 2013). The APOE ε4 allele is specifically linked to a higher likelihood of developing AD and a less favourable reaction to cholinesterase inhibitors, which are routinely employed for AD symptom management (Giri et al., 2016; Van Cauwenberghe et al., 2016; Braak and Del Tredici, 2015; Spillantini et al., 1997; Stewart et al., 1997). For instance, pharmacogenomic research has demonstrated that patients with the APOE ε4 allele may need modified doses or other treatment approaches to achieve the best clinical results. Recent research has emphasized the significance of other genetic variables in AD, including the TREM2 gene, which plays a role in the functioning of microglia and the inflammatory response. TREM2 variants have been linked to a higher likelihood of developing late-onset AD and may impact how individuals respond to anti-inflammatory treatments (Wyss-Coray and Rogers, 2012; Guerreiro et al., 2013; Van Dongen and Van Gool, 2004). Moreover, variations in the BDNF gene, responsible for encoding brain-derived neurotrophic factors, have been associated with cognitive deterioration and the effectiveness of treatment in patients with AD (Wright and Van der Flier, 2014; Lim et al., 2014; Jagust et al., 2018; Venegas and Heneka, 2017). Considering these genetic characteristics, pharmacogenomic methods can enhance treatment strategies and results for people with AD.

### Parkinson’s disease

PD is mainly characterized by the deterioration of dopaminergic neurones in the substantia nigra, resulting in both motor and non-motor symptoms (Irwin et al., 2013). The presence of genetic variants in the CYP2D6 gene, which is responsible for producing an enzyme involved in the metabolism of drugs, has a significant impact on how individuals respond to dopaminergic medications like levodopa and dopamine agonists (Nalls et al., 2019). Individuals with specific CYP2D6 polymorphisms may exhibit variations in the metabolism of various medications, resulting in altered effectiveness and the probability of experiencing adverse reactions (Wang et al., 2006). Pharmacogenomic testing for CYP2D6 enables the customization of treatment programs, guaranteeing that patients are administered the smost suitable and efficient medicine. Additional genetic factors that impact PD include abnormalities in the GBA gene (Thomas et al., 2004). These mutations are linked to Gaucher disease and elevate the likelihood of developing PD. Mutations in the GBA gene can impact how PD patients respond to dopaminergic treatments and their susceptibility to cognitive deterioration (Sidransky et al., 2009; Uddin et al., 2020)). Additionally, variations in the COMT gene, responsible for encoding catechol-O-methyltransferase, can impact how levodopa is metabolized and the likelihood of experiencing motor problems (Vilar Dde et al., 2014; Taymans et al., 2014). Moreover, the discovery of COMT polymorphisms enables targeted adjunct therapies with COMT inhibitors for improved treatment outcomes. Pharmacogenomics also holds promise for more sophisticated interventions through deep brain stimulation (DBS) by evaluating individual genetic responses to dopaminergic treatments (Mehanna et al., 2017). Pharmacogenomics can improve PD management and patient outcomes by using these genetic findings (Beach et al., 2008).

### Huntington’s disease

Huntington’s disease is an inherited neurological ailment that occurs due to increased CAG repeats in the HTT gene. This genetic mutation results in the synthesis of an altered form of the huntingtin protein, which forms clumps and leads to the demise of nerve cells. The genetic foundation of HD presents a distinct objective for pharmacogenomic therapies (Karran and De Strooper, 2016). Genetic profiling can be utilized to forecast the effectiveness of medications intended to decrease the amounts of mutant huntingtin or to regulate its harmful impacts (Ross and Tabrizi, 2011). Pharmacogenomic strategies in HD concentrate on creating treatments that address the fundamental genetic abnormality. Advancements in CRISPR-Cas9 have demonstrated success in reducing toxic proteins in HD models, targeting the HTT gene. Antisense oligonucleotides (ASOs) have been specifically engineered to decrease the activity of the mutant HTT gene, which could potentially decelerate the advancement of the disease (Tabrizi et al., 2019). Furthermore, researchers are investigating the possible use of small molecule inhibitors to regulate the function of the proteasome or autophagy pathways as potential therapies for HD (Gao et al., 2018). Pharmacogenomics can expedite the creation of precise treatments for HD by utilizing genetic knowledge to target the underlying cause.

### Amyotrophic lateral sclerosis (ALS)

Amyotrophic lateral sclerosis (ALS) is a degenerative condition affecting the motor neurones, characterized by a multifaceted genetic makeup—mutations in genes such as SOD1, C9orf72, TARDBP, and FUS cause ALS. Pharmacogenomic methods can enhance the effectiveness of current medications, including riluzole and edaravone, by considering these genetic characteristics. Patients with specific genetic alterations may exhibit differential responses to particular treatments, necessitating alternate therapeutic procedures for those who do not respond favourably (Renton et al., 2014). Recent breakthroughs in ALS research have discovered supplementary genetic variations that impact the likelihood and development of the disease (Mehta et al., 2020). For instance, mutations in the ATXN2 gene, which is connected to spinocerebellar ataxia type 2, have been related to a higher likelihood of developing ALS and could impact the effectiveness of treatment (Elden et al., 2010; Tanzi and Bertram, 2005). Furthermore, alterations in the UBQLN2 gene, responsible for producing ubiquitin-2, have been linked to ALS and frontotemporal dementia (Wesnes and Edgar, 2014; Deng et al., 2011; Snow and Shapiro, 2021; Winblad et al., 2016). In ALS, pharmacogenomics facilitates precise drug selection, particularly with riluzole, where polymorphisms in the ABCB1 gene affect treatment efficacy (Woolley et al., 2020). Pharmacogenomics can enhance the management of ALS and improve therapy outcomes by integrating these genetic findings.

## Progress in Pharmacogenomic tools

### Genome-wide association studies (GWAS)

Genome-wide association studies have played a crucial role in finding genetic variations linked to the way drugs affect neurodegenerative illnesses. By examining the genetic composition of extensive groups of patients, GWAS can reveal prevalent and uncommon genetic variations that impact how individuals react to different therapies. These findings can inform the development of personalized medicines and clinical decision-making (Klein et al., 2005). GWAS have discovered many genomic regions linked to neurodegenerative illnesses and how individuals respond to treatment. For instance, GWAS have shown genetic variations in the LRRK2 gene linked to the likelihood of developing PD and the responsiveness to treatment (Nalls et al., 2019). In addition, GWAS have discovered genetic variations in the CR1 and BIN1 genes that are associated with an increased risk of AD and may also affect how individuals respond to anti-amyloid medications (Lambert et al., 2013; Hardy, 2009; Selkoe and Hardy, 2016). Using GWAS discoveries, Pharmacogenomics can improve the accuracy of treatment approaches for neurodegenerative illnesses.

### Next-generation sequencing (NGS)

Next-generation sequencing (NGS) methods allow for thorough genetic profiling by sequencing the complete genomes or exomes (Johnson et al., 2008). NGS can detect both prevalent and infrequent genetic variations that affect how drugs are processed, their effectiveness, and their safety. This efficient method is progressively becoming more feasible in clinical environments, enabling the development of more accurate and tailored treatment strategies founded on an individual’s distinct genetic makeup (Goodwin et al., 2016). NGS has transformed the study of pharmacogenomics, allowing for the discovery of new genetic variations that impact medication response. NGS has revealed uncommon variations in the TREM2 gene linked to the risk of AD and could impact the effectiveness of anti-inflammatory treatments (Guerreiro et al., 2013). In addition, NGS has detected genetic variations in the GCH1 gene that impact the reaction to levodopa in individuals with PD (Kuilenburg et al., 2016). Pharmacogenomics can offer more precise and individualized treatment suggestions by employing NGS.

### CRISPR-Cas9 is a technique used for gene editing

CRISPR-Cas9 is an innovative gene-editing technique that enables accurate alterations of genomic sequences. This technology is crucial for researching gene-drug interactions and formulating gene-based treatments for neurodegenerative illnesses. CRISPR-Cas9 can rectify genetic flaws or regulate gene expression by focusing on specific genetic mutations, which could lead to personalized therapeutic interventions (Doudna and Charpentier, 2014). CRISPR-Cas9 has been utilized to create gene-based treatments for neurodegenerative illnesses by explicitly targeting the genetic abnormalities responsible for the condition. CRISPR-Cas9 has been used to specifically hinder the expression of the mutant HTT gene in HD mice, resulting in decreased quantities of harmful huntingtin protein and an enhancement in neuronal function (Yang et al., 2020). In addition, the CRISPR-Cas9 system has been used to rectify SOD1 mutations in models of ALS, thereby reinstating standard protein functionality and enhancing the survival of motor neurones (Gaj et al., 2017). Pharmacogenomics can expedite the creation of groundbreaking and tailored treatments for neurodegenerative illnesses by utilizing CRISPR-Cas9.

## Application of pharmacogenomics in clinical practice

### Alzheimer’s disease

Pharmacogenomic testing can analyze genes such as APOE and BDNF to determine the appropriate selection and dosage of cholinesterase inhibitors and NMDA receptor antagonists in AD (Oddo and LaFerla, 2006; Miller and Seeley, 2013). Patients with the APOE ε4 allele may experience improved therapeutic results using alternative medicines or modified dosages. Genetic testing to create individualized treatment approaches can enhance the control of symptoms and decelerate the advancement of AD (Yeung and Wong, 2020; Timmerman et al., 2020; Masters et al., 2015). Pharmacogenomics can provide valuable information for utilizing new therapeutic strategies in AD (Maki and Holsinger, 2010; Oddo and LaFerla, 2006). For example, using monoclonal antibodies or small molecule inhibitors to target amyloid-beta and tau pathology may significantly impact patients with specific genetic profiles, as Cummings et al., 2019 suggested (Zheng and Koo, 2011; Zhang et al., 2007; Yan and Stern, 2014; Trojanowski and Lee, 2000; Mattsson et al., 2011; Trojanowski and Lee, 2005; Wang and Mandelkow, 2016; Zhou and Lee, 2011). In addition, pharmacogenomic knowledge can be used to direct the administration of neuroprotective substances, such as antioxidants and anti-inflammatory medications, to decelerate disease advancement and enhance cognitive abilities (Querfurth and LaFerla, 2010; Aisen et al., 2016; Panza et al., 2006; Yang et al., 2020). Pharmacogenomics can improve the accuracy and effectiveness of AD treatments by incorporating genetic testing into clinical practice (Cummings et al., 2015; O’Brien, and Wong, 2011).

### Parkinson’s disease

Genetic testing can identify polymorphisms in CYP2D6, COMT, and other essential genes. This information can be used to customize dopaminergic therapy for individuals with Parkinson’s disease. Clinicians can optimize drug dosages and choose the most suitable treatments to reduce side effects and improve effectiveness by comprehending an individual’s genetic profile (Mammen and Fernandez, 2009). Implementing a customized strategy can significantly enhance the wellbeing of individuals with PD (Kang et al., 2020; Okun, 2012). Pharmacogenomics can also provide valuable insights for the use of modern treatments in PD, such as DBS and gene therapy. Genetic testing can determine which patients are more likely to have positive effects from deep brain stimulation (DBS) by evaluating their responsiveness to dopaminergic medications (Mehanna et al., 2017). In addition, gene therapy methods that focus on particular genetic alterations, such as the use of AAV to deliver GDNF or Nurr1, have the potential to offer neuroprotection and enhance motor function in individuals with Parkinson’s disease (Axelsen and Woldbye, 2018). By integrating pharmacogenomic knowledge, medical professionals can enhance treatment approaches and results for PD patients.

### Huntington’s disease

Targeted medicines, such as antisense oligonucleotides (ASOs) and small-molecule inhibitors, can be customized to match the genetic profiles of individuals with Huntington’s disease (HD). Genetic testing can ascertain the people likely to derive advantages from these medications, enabling a more individualized and efficacious strategy for managing HD. This technique aims to decrease the production of mutant huntingtin protein and alleviate its detrimental impact on neurones (Tabrizi et al., 2019). Pharmacogenomics can also guide using additional therapeutic strategies for Huntington’s disease, including neuroprotective medicines and treatments to alleviate symptoms. Genetic testing can determine the appropriate use of drugs that affect neurotransmitter systems, including dopamine antagonists and glutamate modulators, to enhance both motor and mental symptoms (Frank, 2014). In addition, the use of pharmacogenomic information can aid in creating combination medicines that specifically target various pathways implicated in the development of Huntington’s disease (Smith et al., 2014). Pharmacogenomics can improve the accuracy and effectiveness of HD treatments by incorporating genetic testing into clinical practice.

### Amyotrophic lateral sclerosis (ALS)

Genetic factors should be considered to optimize the utilization of current medications for ALS, such as riluzole and edaravone, through pharmacogenomic insights. Patients with specific genetic alterations may exhibit enhanced responsiveness to particular medicines, but different therapeutic approaches may be necessary for others. Genetic testing can guide the selection and dosage of these medications, leading to enhanced therapeutic results and a decrease in the likelihood of adverse side effects (Taylor et al., 2016). Pharmacogenomics can provide valuable insights into the application of innovative treatment methods in ALS, including gene therapy and stem cell therapy (Guerini et al., 2015). One possible approach to address the C9orf72 mutation is to use ASOs or CRISPR-Cas9 to target it specifically. This can decrease the creation of harmful RNA foci and dipeptide repeat proteins, which may slow the disease’s progression (DeJesus-Hernandez et al., 2011). In addition, stem cell therapy methods that administer neurotrophic factors or substitute impaired motor neurones can offer neuroprotection and enhance motor function in individuals with ALS By integrating pharmacogenomic knowledge, medical practitioners can enhance treatment approaches and results for ALS patients.

## Obstacles and prospects for the future

### Ethical and legal factors to be taken into account

Implementing pharmacogenomics raises several ethical and legal concerns, such as genetic privacy, informed consent, and the possibility of discrimination based on genetic data. Guaranteeing fair access to personalized treatment and safeguarding patient privacy is crucial for the success of pharmacogenomics. In order to promote the widespread adoption of pharmacogenomic techniques, policymakers and healthcare professionals need to address these issues and work towards building trust (Burke et al., 2010). In order to tackle these difficulties, it is imperative to establish solid regulatory frameworks and rules to control the utilization of genetic information in clinical practice. These frameworks must guarantee that genetic testing is carried out in an ethically sound manner, with enough measures in place to respect patient privacy and prevent discrimination. In addition, it is crucial to promote public education and participation to increase understanding of pharmacogenomics’s advantages and potential drawbacks. This will facilitate informed decision-making and acceptance among patients and healthcare providers (McGuire et al., 2008; Strobel et al., 2004).

### Data integration and analysis

Integrating genetic, clinical, and pharmacological data is a substantial barrier (Galvin et al., 2001; Pardridge, 2005). However, it is crucial for the advancement of pharmacogenomics. Advanced bioinformatics tools and machine learning techniques are essential for effectively organizing and analyzing this intricate data. These technologies can aid in identifying patterns and correlations, enabling the creation of personalized treatment programs based on a thorough comprehension of an individual’s genetic and clinical profile (Kohane, 2015). Effective data integration requires interdisciplinary collaboration among geneticists, doctors, bioinformaticians, and data scientists. In addition, it is crucial to build standardized protocols and data-sharing frameworks to enable the seamless transmission of genetic and clinical information among various healthcare settings (Zucker and Regehr, 2002). Pharmacogenomics can optimize the use of genetic information to enhance patient care by utilizing advanced data analytics and promoting collaboration (Johnson et al., 2013).

### Customized clinical trials

**FIGURE 1:**
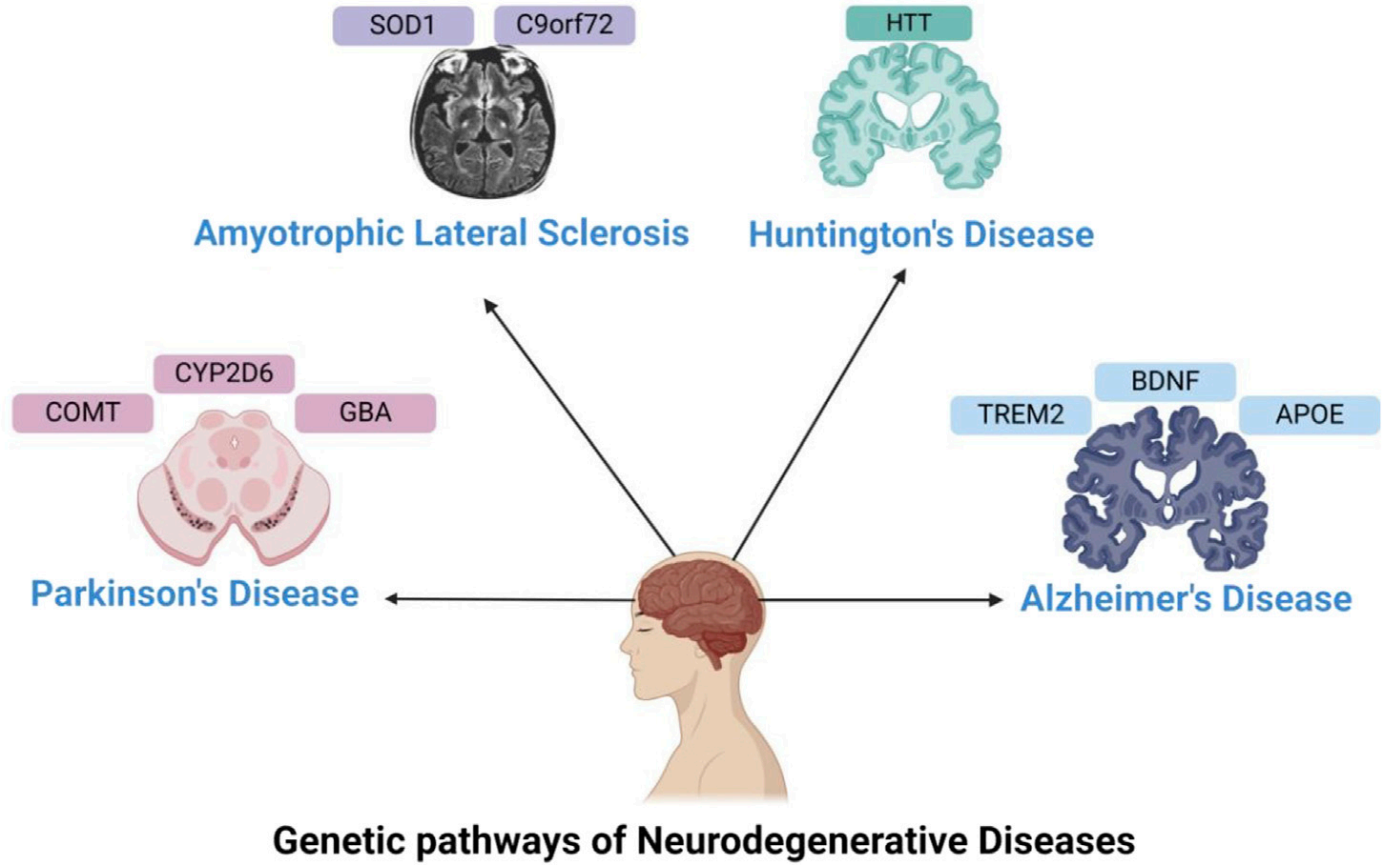
Genetics in Neurodegenerative Disorders—This figure illustrates the genes involved in neurodegenerative disorders, such as APOE, TREM2, and BDNF for Alzheimer’s disease; CYP2D6, GBA, and COMT for Parkinson’s disease; HTT for Huntington’s disease; and SOD1 and C9orf72 for ALS.

**FIGURE 2:**
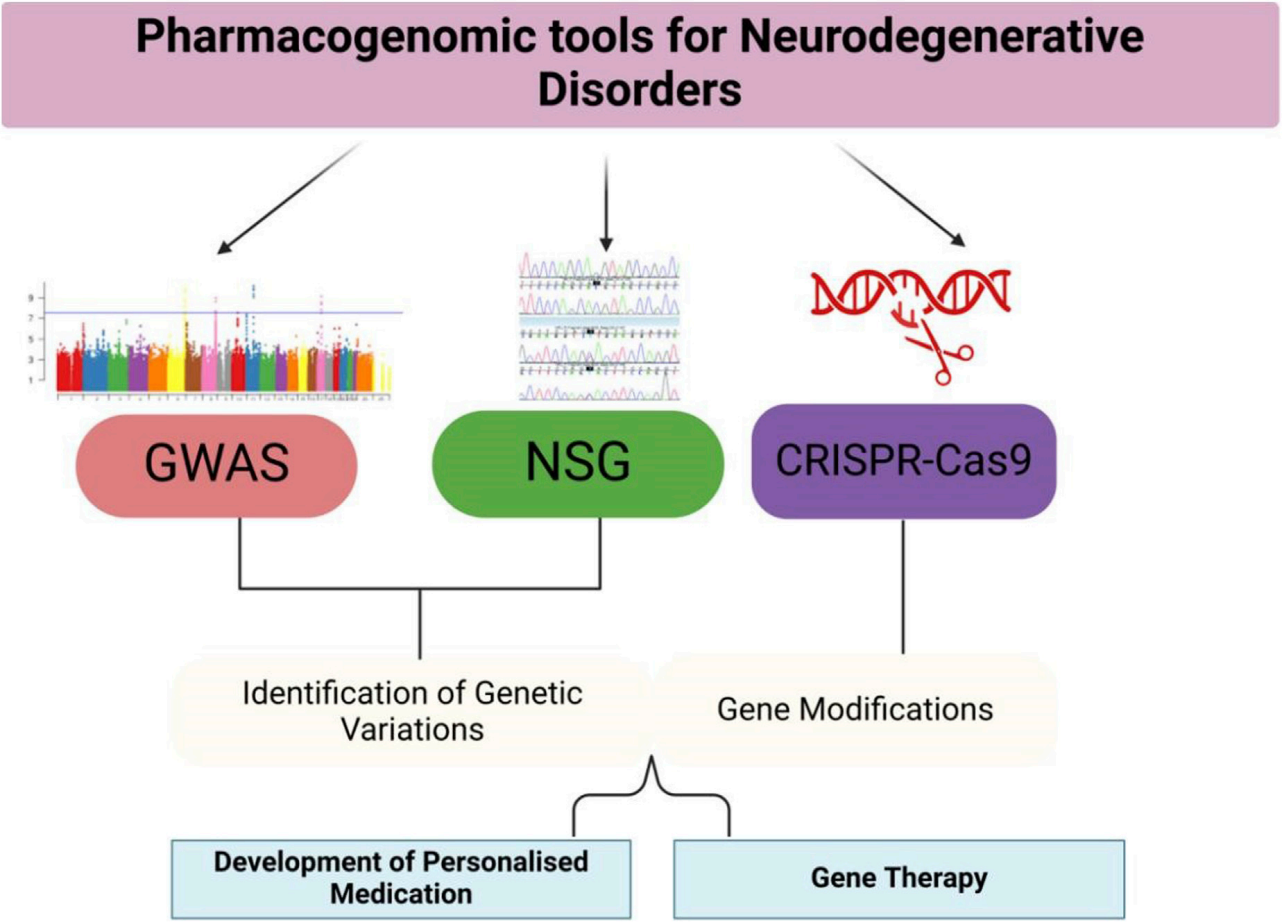
Pharmacogenomic Tools and Applications—This figure provides an overview of the pharmacogenomic tools used in the study and treatment of neurodegenerative disorders. GWAS, NGS, and CRISPR-Cas9 are applied to identify genetic variations, develop personalized treatments, and modify genes. The figure also illustrates the applications of these tools in clinical settings, such as tailored drug prescriptions and gene therapies.

Conventional clinical trial designs may not be appropriate for pharmacogenomic research, as they frequently overlook the genetic diversity among participants. Researchers are currently investigating adaptive trial designs and N-of-1 trials, explicitly targeting individual patients, to assess the effectiveness of personalized treatments. These novel methodologies can offer more pertinent and precise data, directing the advancement and execution of pharmacogenomic treatments (Woodcock and LaVange, 2017). Adaptive trial designs permit adjustments to the trial protocol based on interim data analysis, enabling researchers to modify treatment regimens and optimize patient results in real time. In contrast, N-of-1 trials specifically target individual patients and use multiple assessments to evaluate the effectiveness of various treatments. These methods can offer helpful knowledge about the effectiveness and safety of personalized treatments, making it easier to create customized treatment strategies for neurodegenerative diseases (Lillie et al., 2011). The results of Genetic variants and their impact on drug response in neurodegenerative diseases are shown in Table 1, including pathways and genes in Figure 1. Different pharmacological tools are shown in Table 2 and Figure 2.

**TABLE 1:** Genetic variants and their impact on drug response in neurodegenerative diseases.

Gene	Disease	Genetic variant	Impact on drug response	References
APOE	Alzheimer’s Disease	ε4 allele	Linked to higher risk and less favourable response to cholinesterase inhibitors	Giri et al. (2016), Van Cauwenberghe et al. (2016), Roberson and Mucke (2006), Hardy (2017), Lovestone and Harrington (2006); Reitz et al. (2011), Ringman and Coppola (2013)
TREM2	Alzheimer’s Disease	Variants	Affects response to anti-inflammatory treatments	Ulrich and Holtzman (2016), Wang et al. (2007), Guerreiro et al. (2013), Munoz and Ammit (2010), Naj et al. (2011), Nixon and Yang (2011), Schneider et al. (2014), Sultana and Butterfield (2010), Xie et al. (2010)
BDNF	Alzheimer’s Disease	Val66Met	Associated with cognitive decline and treatment effectiveness	Lim et al., (2014), Salloway et al. (2014), Sindi et al. (2017), Solomon et al. (2014), Yao et al. (2011), Zetterberg and Blennow (2013)
CYP2D6	Parkinson’s Disease	Polymorphisms	Influences metabolism and effectiveness of dopaminergic medications	Nalls et al. (2019), Trinh and Farrer (2013)
GBA	Parkinson’s Disease	Mutations	Linked to higher risk and impacts response to dopaminergic treatments	Sidransky et al. (2009), Schneider and Obeso (2015)
COMMENT	Parkinson’s Disease	Variants	It affects levodopa metabolism and the likelihood of motor complications	Vilar Dde et al. (2014)
HTT	Huntington’s Disease	CAG repeats	Determines severity and progression of disease; targets for antisense oligonucleotides	Ross and Tabrizi (2011), Rodríguez et al. (2009)
SOD1	ALS	Mutations	Impacts effectiveness of riluzole and edaravone	Renton et al. (2014)
C9orf72	ALS	Repeat expansions	Linked to ALS-FTD; potential target for ASOs	DeJesus-Hernandez et al. (2011), Renton et al. (2011)

**TABLE 2:** Pharmacogenomic tools and applications in neurodegenerative disease research.

Tool	Description	Application	Examples	References
Genome-Wide Association Studies (GWAS)	Identifies genetic variants across the genome associated with diseases and drug responses	Finding genetic markers for disease risk and drug response	LRRK2 variants in Parkinson’s disease; CR1 and BIN1 variants in Alzheimer’s disease	Nalls et al. (2019), Lambert et al. (2013), Smith et al. (2006)
Next-Generation Sequencing (NGS)	Sequencing entire genomes or exomes to identify genetic variations	Comprehensive genetic profiling	Identification of TREM2 variants affecting anti-inflammatory treatment in Alzheimer’s disease	Guerreiro et al. (2013), Goodwin et al. (2016), Venigalla et al. (2015)
CRISPR-Cas9	Gene-editing technology to modify specific genes	Investigating gene-drug interactions and developing gene-based therapies	Targeting HTT gene in Huntington’s disease models; correcting SOD1 mutations in ALS models	Doudna and Charpentier (2014), Yang et al. (2017), Gaj et al. (2017)
Antisense Oligonucleotides (ASOs)	Short DNA or RNA molecules designed to bind to specific mRNA sequences	Reducing expression of mutant genes	ASOs targeting mutant HTT gene in Huntington’s disease; ASOs for C9orf72 mutation in ALS	Tabrizi et al. (2019), DeJesus-Hernandez et al. (2011)
Biomarker Identification	Detection of biological markers indicative of disease state or treatment response	Monitoring disease progression and treatment efficacy	APOE genotyping for Alzheimer’s disease risk; GBA mutation analysis in Parkinson’s disease	Giri et al. (2016), Szatmari and Frenyo (2021), Sidransky et al. (2009), Drogan et al. (2015), Jack et al. (2010), Toledo and Arnold (2013)

### Pharmacogenomics Pathways in neurodegenerative diseases

Pharmacogenomics is becoming a crucial tool in the treatment of neurodegenerative diseases, allowing for more personalized and precise therapies. In AD, PD, HD, and ALS, genetic variations significantly influence drug responses, highlighting the need for personalized treatment approaches (Swen et al., 2011). However, translating these findings into clinical practice presents challenges, particularly in identifying reliable genetic markers and managing polygenic influences on disease and treatment outcomes.

#### Pharmacogenomics pathway in Alzheimer’s disease

AD is primarily influenced by genetic risk factors, particularly the APOE gene, which has significant pharmacogenomic implications (Goedert and Spillantini, 2006). The APOE-ε4 allele is associated with an increased risk of AD and has been shown to influence responses to cholinesterase inhibitors, such as donepezil and rivastigmine, as well as memantine, which is used to moderate symptoms of moderate to severe AD (Vilar and Llorens-Martin, 2021; Cacabelos, 2020; Lam et al., 2017; Serrano-Pozo et al., 2011). For example, APOE-ε4 carriers often show less favorable responses to these drugs, leading to the need for personalized therapeutic approaches (Cacabelos, 2008). Additionally, variations in the CYP2D6 enzyme, responsible for drug metabolism, have been associated with differential responses to these medications. Poor metabolizers of CYP2D6 may experience more pronounced side effects from cholinesterase inhibitors, while ultra-rapid metabolizers may require higher doses to achieve therapeutic effects (Zhou and Lee, 2011; Youdim and Buccafusco, 2005).

#### Pharmacogenomics pathway in Parkinson’s disease

PD treatment is highly dependent on dopamine replacement therapies, such as levodopa and dopamine agonists. Genetic variants in enzymes such as CYP2D6 influence the metabolism of dopamine agonists (Mizutani et al., 2021). Poor metabolizers may experience enhanced drug toxicity, while ultra-rapid metabolizers often require higher doses for effective treatment (Zhou et al., 2021). Moreover, polymorphisms in the COMT gene, which encodes the enzyme catechol-O-methyltransferase, play a role in the metabolism of levodopa. The Val158Met variant of COMT affects the breakdown of dopamine, influencing the response to levodopa and the need for adjunct therapies like COMT inhibitors (Bialecka et al., 2007). Familial PD associated with LRRK2 and GBA mutations also illustrates how pharmacogenomics can influence treatment strategies. For instance, GBA mutation carriers often have a more aggressive disease course and may benefit from early initiation of neuroprotective treatments (Gan-Or et al., 2015).

#### Pharmacogenomics pathway in Huntington’s disease

HD is caused by an expansion of the CAG repeat in the HTT gene, and gene-based therapies targeting HTT are in development to reduce mutant HTT protein production (Southwell et al., 2018). Pharmacogenomic approaches in HD are primarily focused on symptomatic treatment, such as managing chorea with tetrabenazine and deutetrabenazine. Variations in CYP2D6 affect the metabolism of these drugs, with poor metabolizers at increased risk for adverse effects (Walker et al., 2015; Robins Wahlin et al., 2012). Additionally, gene silencing therapies targeting the HTT gene, such as antisense oligonucleotides (ASOs) and RNA interference (RNAi), are in clinical trials and represent a promising area where pharmacogenomics can play a significant role (Southwell et al., 2018).

#### Pharmacogenomics pathway in Amyotrophic lateral sclerosis

In ALS, genetic mutations in genes like C9orf72, SOD1, and TARDBP play critical roles in disease pathogenesis. Gene therapies targeting SOD1 mutations are being developed to suppress mutant protein expression (Miller and Schademan, 2020). Pharmacogenomic approaches in ALS also focus on the variability in drug response, such as with riluzole and edaravone. For example, polymorphisms in the ABCB1 gene, which encodes P-glycoprotein, can affect the absorption and efficacy of riluzole (Woolley et al., 2020).

## Discussion

Pharmacogenomics, the study of how genes affect an individual’s response to drugs, is playing an increasingly important role in the treatment of neurodegenerative disorders, such as AD, PD, HD and ALS (Seitz et al., 2010; Sierksma et al., 2020). This field of personalized medicine has the potential to significantly improve treatment outcomes by tailoring therapies to an individual’s genetic makeup, thereby optimizing drug efficacy and minimizing adverse effects (Hyman et al., 2012; Mackenzie et al., 2010). The growing application of genetic insights in the treatment of neurodegenerative diseases is rooted in the identification of genetic markers that influence how patients metabolize and respond to medications (Weiner et al., 2015). For instance, in Alzheimer’s disease, variations in the APOE gene are associated with differences in response to certain drugs, such as cholinesterase inhibitors, which are commonly prescribed to slow cognitive decline (Ray et al., 2011). Similarly, genetic polymorphisms in enzymes such as CYP2D6 and CYP2C19 affect drug metabolism in PD patients, influencing the efficacy and tolerability of dopamine agonists and other therapies (Haass and Selkoe, 2007; Tobe and Jurisic, 2018). In Huntington’s disease, the identification of genetic modifiers of disease progression has opened new avenues for therapeutic intervention and drug development.

However, translating these genetic findings into clinical practice presents several challenges. One major hurdle is the polygenic nature of neurodegenerative disorders, where multiple genes contribute to disease susceptibility and drug response. This complexity complicates the identification of single genetic markers that can reliably predict treatment outcomes. Additionally, gene-environment interactions, such as the role of lifestyle factors, further influence disease progression and treatment efficacy, making it difficult to disentangle the genetic determinants of drug response from other contributing factors. Ethical considerations are also central to the growing role of pharmacogenomics in neurodegenerative disorders. Genetic privacy is a critical concern, as patients may be reluctant to undergo genetic testing if they fear that their genetic information could be misused. Furthermore, access to pharmacogenomic testing is not uniform across populations, with underserved communities often lacking access to advanced genetic testing and personalized treatment options. Addressing these disparities is essential to ensure that the benefits of pharmacogenomics are accessible to all patients, regardless of socioeconomic background.

## Conclusion

Pharmacogenomics has the potential to revolutionize the treatment of neurodegenerative disorders by enabling more personalized and effective therapeutic strategies. The identification of genetic markers that influence drug response is paving the way for tailored treatments that improve patient outcomes and reduce adverse drug reactions. However, significant challenges remain, including the complexity of polygenic influences, the interplay between genetic and environmental factors, and the ethical considerations surrounding genetic testing and data privacy. Ongoing research is essential to uncover new genetic markers and refine our understanding of how genetics can be leveraged to optimize therapy for neurodegenerative diseases. Collaborative efforts between geneticists, clinicians, and bioethicists are critical to overcoming current limitations and ensuring that the benefits of pharmacogenomics are equitably distributed across patient populations. As research advances and new technologies emerge, pharmacogenomics offers a pathway to more personalized, effective, and ethical healthcare solutions for neurodegenerative diseases.

## Abbreviations

AD, Alzheimer’s Disease; PD, Parkinson’s Disease; HD, Huntington’s Disease; ALS, Amyotrophic Lateral Sclerosis; APOE, Apolipoprotein E; TREM2, Triggering Receptor Expressed on Myeloid Cells 2; BDNF, Brain-Derived Neurotrophic Factor; CYP2D6, Cytochrome P450 2D6; GBA, Glucosylceramidase Beta; COMT, Catechol-O-Methyltransferase; HTT, Huntingtin; SOD1, Superoxide Dismutase 1; C9orf72, Chromosome 9 Open Reading Frame 72; GWAS, Genome-Wide Association Studies; NGS, Next-Generation Sequencing; CRISPR, Clustered Regularly Interspaced Short Palindromic Repeats; ASOs, Antisense Oligonucleotides.
